# Strategy for pattern recognition‐driven optical chemosensing based on polythiophene

**DOI:** 10.1002/smo.20240001

**Published:** 2024-06-01

**Authors:** Binduja Mohan, Yui Sasaki, Tsuyoshi Minami

**Affiliations:** ^1^ Institute of Industrial Science The University of Tokyo Tokyo Japan; ^2^ JST PRESTO Kawaguchi Saitama Japan

**Keywords:** chemosensor array, pattern recognition, polymers, polythiophene, sensors

## Abstract

Polythiophenes (PTs) with flexible backbones possess inherent polymer behaviors, including molecular wire effects and dynamic structural changes in π‐conjugated systems. The chemical sensing at the functionalized side chains can manipulate such polymer characteristics, resulting in various optical patterns depending on the analyte structures and their concentrations. The unique optical patterns derived from polymer properties contribute to group categorization over a wide concentration range for pattern recognition. This review aims to provide a concise overview of the potential of PT chemosensor arrays using actual sensing examples in environmental monitoring, medical diagnostics, and food analysis. Furthermore, this review summarizes the methodologies that use polymer gels to realize practical chemosensor array chips for onsite analysis.

## Graphical abstract

Polythiophenes with flexible backbones possess inherent polymer behaviors including molecular wire effects and dynamic structural changes in π‐conjugated systems, which contribute to optical pattern recognition of various analytes at wide concentration ranges.

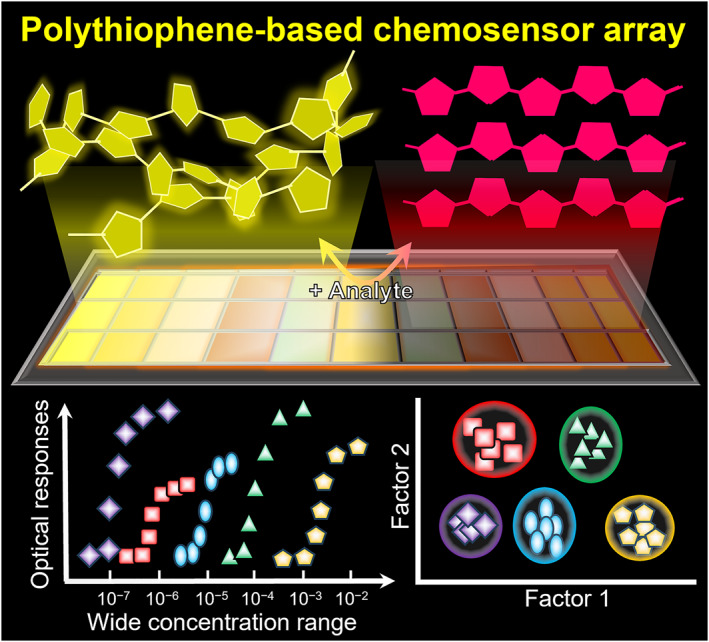

## 1 INTRODUCTION

**FIGURE 1:**
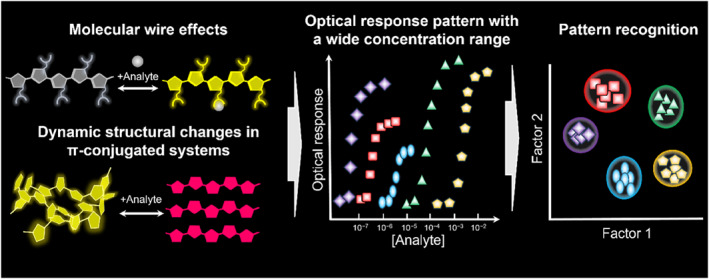
Representation of pattern recognition‐driven chemosensing based on polythiophene derivatives with inherent molecular wire effects and dynamic structural changes in π‐conjugated systems upon detecting various analytes at wide concentration ranges.

Optical chemosensor arrays are essential analytical devices based on colorimetric and fluorescence properties for the simultaneous qualitative and quantitative detection of multiple analytes. The feasibility of pattern recognition is attributed to the cross‐reactivity of chemosensors, which provide fingerprint‐like response patterns in chemical sensing.^[1]^ Depending on the analyte structures and concentrations, various optical responses are analyzed using pattern recognition algorithms.[^2^, ^3^, ^4^] To date, extensive efforts have been dedicated to the development of various chemosensor arrays using small molecules,[^5^, ^6^] π‐conjugated polymers,[^7^, ^8^, ^9^] and nanomaterials,[^10^, ^11^, ^12^] and their pattern recognition abilities have been evaluated in areas such as bioapplications, environmental monitoring, and food chemistry. Conventional chemosensor arrays typically incorporate multiple chemosensors with different receptors, reporters, or combinations of both components.^[6]^ The assembly of chemosensor elements with different optical properties is necessary to obtain a fingerprint‐like response for effective pattern recognition. However, some chemosensors used for an array cause misclassification and/or a decrease in the response spaces among each cluster, originating from the low discriminatory abilities of the chemosensors.[^13^, ^14^] A sophisticated chemosensor design provides various optical response patterns that can be used as sufficient datasets for pattern recognition, even with a small number of sensor elements. This approach leads to the concept of a single chemosensor‐driven pattern recognition.[^14^, ^15^] In this regard, molecular self‐assemblies have been employed as driving forces to obtain various optical patterns derived from assembly and disassembly in chemical sensing,[^5^, ^16^] and the applicability of this concept has been revealed by various sensing applications based on pattern recognition by single chemosensors.[^14^, ^15^] The strategy of chemosensor design based on molecular self‐assemblies allows the discrimination of analyte structures in a categorized group (e.g., carboxylates and phosphates) at a certain concentration; however, group categorization of various analytes over a wide range of concentrations is still challenging. Among the chemosensor platforms, π‐conjugated polymers possess unique features, including high sensitivity owing to molecular wire effects and selectivity facilitated by numerous binding sites in the functionalized side chains.[^17^, ^18^, ^19^, ^20^] Polythiophenes (PTs) are widely employed as chemosensor skeletons because of their flexible backbones, which provide various optical changes derived from the polymer dynamics between the random coils and planarized structures (and further aggregation).[^21^, ^22^, ^23^, ^24^, ^25^] The functional groups at the 3‐ and/or 4‐positions along the thiophene backbone play significant roles in tuning the polymer behavior by detecting chemical and physical stimuli.[^26^, ^27^] In particular, the polymer behavior of PT‐based chemosensors depends significantly on the polarity of the sensing environment and the binding affinities with the analytes.[^28^, ^29^] In contrast to other π‐conjugated polymers with rigid backbones, such as poly(*p*‐phenylene ethynylene) and polyfluorene, PT‐based fluorescent chemosensors with flexible backbones can show significant shifts in emission wavelength by analyte capture.^[30]^ Thus, functionalized PTs endowed with polymer dynamics and molecular wire effects are favorable chemosensors for obtaining enriched optical responses for group categorization of various analytes at different concentrations, albeit with a small number of sensor elements (vide infra) (Figure 1). In this review, we focused on the design of optical PT derivatives for pattern‐recognition‐driven chemosensing beyond conventional chemosensor arrays.

## 2 GENERAL SCHEME FOR PATTERN RECOGNITION

**FIGURE 2:**
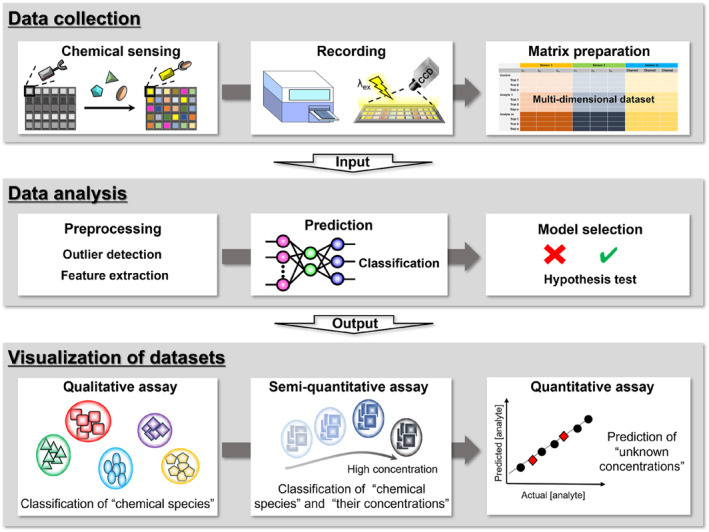
Representation of the concept of multi‐sensing through the utilization of chemosensor arrays and pattern‐recognition techniques.

This review article introduces solution‐based PT chemosensor arrays (Section 3) and solid‐state PT chemosensor devices (Section 4) for onsite analyses. Herein, general sensing and data analysis are summarized for solution‐ and solid‐state chemosensor devices. Pattern recognition is systematically categorized into three stages: (1) data collection, (2) data analysis, and (3) visualization of datasets (Figure 2).[^2^, ^6^] In the initial phase, spectrophotometers are used to record various absorption or fluorescence changes in each chemosensor solution upon adding the analytes. Therefore, a dataset of solution‐based arrays contains absorbance and fluorescence intensities corresponding to each wavelength. Meanwhile, optical changes in array chips embedded with chemosensors can be captured using recordable equipment such as flatbed scanners, digital cameras, or smartphones. Subsequently, imaging analysis is applied to the digital images to extract their color intensities. The outcomes within the dataset are characterized by the color intensities corresponding to each channel.^[9]^ In the data analysis phase, these multidimensional datasets are treated using statistical techniques for pre‐data processing. For example, the Student's *t*‐test eliminates outliers from the obtained dataset. In addition, analysis of variance (ANOVA) is performed to indicate variables in the optical properties that significantly contributes to the classification process.^[31]^ These pre‐processing techniques improve classification accuracy by removing the noise factors identified within the original datasets. The treated datasets, referred to as inset datasets, are subsequently subjected to pattern recognition for the classification of chemical species at varying concentrations (qualitative or semi‐quantitative detection) or the prediction of unknown concentrations (quantitative detection). Pattern recognition techniques in chemosensing are categorized into two types: supervised methods, including linear discriminant analysis (LDA),[^2^, ^3^] support vector machine (SVM),[^32^, ^33^] and artificial neural network (ANN),[^14^, ^34^, ^35^, ^36^, ^37^] and unsupervised methods, such as principal component analysis (PCA) and hierarchical clustering analysis (HCA).[^2^, ^3^] For example, LDA reduces the dimensionality of information‐rich inset data, and visualizes them as two‐ or three‐dimensional output results.^[6]^ This method is performed using leave‐one‐out cross‐validation, which can be used to assess the quality of the sensing abilities of the chemosensors. In addition, SVM and ANN, as potent machine learning tools, enable building linear calibration lines, even for nonlinear response patterns. Thus, these methods can be applied to real sample analyses, particularly for complex samples such as environmental water, biological, and food samples. The precision of the calibration model and its predictive capability are assessed using the root mean square errors of calibration (RMSEC) and prediction (RMSEP) values.^[32]^ In contrast to supervised methods, unsupervised methods can indicate the quality of the inset data based on the tendency of the output results (e.g., the position of distributed clusters). This systematic approach based on pattern recognition techniques provides a potent framework for high‐throughput analysis using abundant chemical information.

## 3 SINGLE CHEMOSENSOR‐DRIVEN PATTERN RECOGNITION

The inherent polymer behavior, including molecular wire effects and changes in π‐conjugated systems, provides variation in optical properties depending on the structural geometries of analytes and their concentrations.[^29^, ^31^] This section highlights a chemosensor design strategy for pattern recognition using single PT derivatives.

**FIGURE 3:**
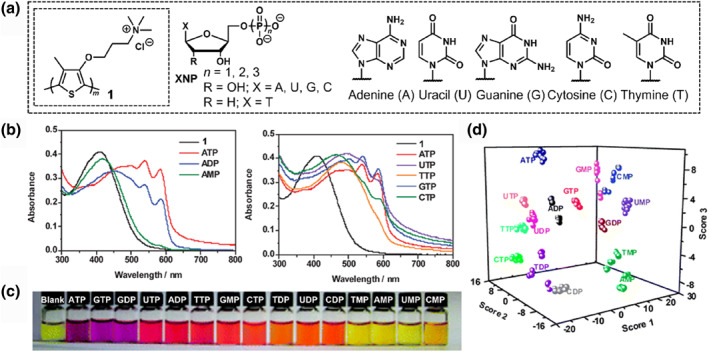
(a) Chemical structures of **1** and the target nucleotides (XNPs). (b) UV‐vis absorption spectra of **1** (0.1 mM) upon the addition of (left) ATP, ADP, and AMP, and (right) ATP, UTP, TTP, GTP, and CTP in 4‐(2‐hydroxyethyl)‐1‐piperazineethanesulfonic acid (HEPES) buffer (pH 7.4). (c) Visual representation of the color changes of **1** (0.1 mM) upon adding nucleotides (0.4 mM) in the aqueous media. (d) LDA canonical score plot for the qualitative detection of 15 nucleotides at three different concentrations. *Source*: Reproduced with permission from [38] 2009 The Royal Society of Chemistry.

With a focus on the development of a single PT derivative for pattern recognition, the collaborative efforts of Li and Shi research groups designed a water‐soluble PT derivative for the discrimination of 15 nucleotide phosphates (XNPs, where X = adenine (A), uracil (U), thymine (T), guanine (G), cytosine (C), and NPs = nucleophosphates [MP = monophosphate, DP = diphosphate, and TP = triphosphate]) (Figure 3a).^[38]^ The discrimination of nucleotides is essential in biological reactions and metabolism;^[39]^ however, the strategy for chemosensor design has not been fully established because of the complicated analyte structures with differences in nucleobase structures and numbers of phosphate moieties.[^24^, ^40^, ^41^, ^42^, ^43^, ^44^, ^45^, ^46^] In this study, water‐soluble PT functionalized with a quaternary ammonium group (**1**) contributed to the formation of ionic self‐assemblies with the phosphate groups of the analytes.[^24^, ^47^] The polymer structure can bind to the analytes through electrostatic and hydrophobic interactions and π‐π stacking, which provides colorimetric changes originating from conformational changes or the aggregation of conjugated backbones.^[24]^ As shown in Figure 3b, the optical alterations corresponded to differences in the number of phosphate moieties and nucleobase structures, which contributed to obtaining various colorimetric patterns to discriminate the nucleotides (Figure 3c). In this regard, an increase in the number of phosphate groups causes the aggregation of polymer wires, resulting in Rayleigh scattering at longer wavelengths.^[48]^ Therefore, the optical response profiles revealed that the interplay between the hydrophilic and hydrophobic interactions influenced the planarization of the PT backbones and further aggregation.^[38]^ Hence, LDA was performed to assess the potential of **1** for nucleotide identification using various colorimetric profiles derived from a single chemosensor. The original data, comprising eight variables of the UV‐vis absorption spectra without pre‐processing, were analyzed using the Statistics and Statistical Graphics software package (SYSTAT). The output resulted in a three‐dimensional score plot (Figure 3d) where all 15 nucleotides appeared as well‐separated groups, demonstrating 100% accurate discrimination. This demonstration clarified the discriminatory ability of the PT derivative for nucleotides, resulting in various spectral patterns through different interaction mechanisms, even in a single chemosensor.

**FIGURE 4:**
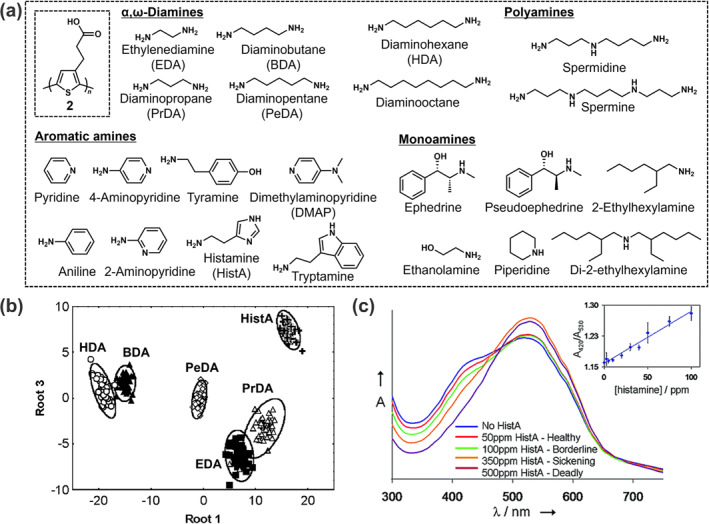
(a) Chemical structures of **2** and target amines. (b) LDA canonical score plot for the qualitative detection of six amines in HEPES buffer (pH = 7.4). [**2**] = [amine] = 1 mM. (c) Colorimetric response of **2** to HistA in the real sample of tuna fish. *Source*: Reproduced with permission from [49] 2007 American Chemical Society.

As shown above, the analyte‐induced aggregation phenomena cause the formation of multiple assemblies with analytes, leading to various optical patterns. To utilize the unique characteristics of PTs, Lavigne et al. designed a carboxy‐group‐attached PT derivative (**2**) for the detection of a broad range of biogenic amines, including aromatic and aliphatic mono‐, di‐, and polyamines (Figure 4a).^[49]^ Target biogenic amines and thier derivatives are crucial markers involving cancer, bacterial infection, and food poisoning, owing to their association with rapid cell proliferation.[^50^, ^51^, ^52^, ^53^] Therefore, the detection of amines is essential for food assessment and diagnosis. In this detection mechanism, interactions between the PT derivative (**2**) and the amines induce conformation changes in the backbones and further aggregation derived from π‐π stacking among the backbones. Hence, various colorimetric responses could be obtained by forming multiple assemblies of a single PT derivative (**2**) and amines. In this assay, LDA was employed for the qualitative assessment of six representative amines in the target list (i.e., diaminohexane [HDA], diaminobutane [BDA], diaminopentane [PeDA], ethylenediamine [EDA], diaminopropane [PrDA], and histamine [HistA]). Figure 4b shows 99% classification rate for six amines with 95% confidence ellipsoids, whereas a decrease in classification accuracy (97%) was observed for 22 amines. The misclassification was derived from the insufficient discriminatory ability of **2** for the diastereomeric structures of ephedrine and pseudoephedrine. Given that HistA is a crucial food marker for fish poisoning,[^54^, ^55^, ^56^] this polymer chemosensor (**2**) was further used to detect varying concentrations of HistA in tuna fish samples (Figure 4c). The concentration‐dependent colorimetric changes suggest the potential of the described approach using the polymer for early and precise detection of spoilage in food samples.

**FIGURE 5:**
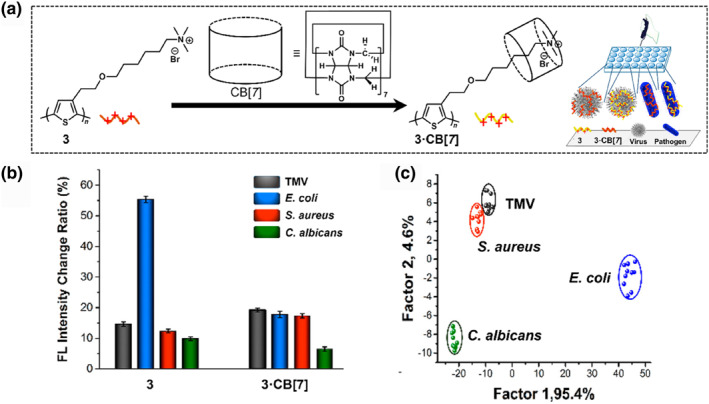
(a) Schematic representation of the design of the self‐assembled system using **3** and CB[7] for detecting viruses and pathogens. (b) Presentation of the fluorescence response histogram **3** (20 μM) and **3**·CB[7] (20 μM) to various microorganisms and viruses in phosphate‐buffered saline. [TMV] = 0.15 and 0.2 mg/mL, [*E. coli*] = 7.5 × 10^7^ and 1.0 × 10^8^ cfu/mL, [*S. aureus*] = 9 × 10^7^ and 1.2 × 10^8^ cfu/mL, and [*C. albicans*] = 4 × 10^7^ and 5 × 10^7^ cfu/mL. (c) LDA canonical score plot for discriminating the viruses and pathogens. *Source*: Reproduced with permission from [66] 2018 American Chemical Society.

π‐Conjugated polymers have gained attention in the field of biological sensing owing to their ability to amplify optical signals,[^57^, ^58^, ^59^, ^60^] which has been further expanded to the detection of pathogenic microorganisms. In conventional sensor designs, charged conjugated polymers have been utilized for the detection and identification of various bacteria,[^60^, ^61^, ^62^, ^63^] whereas their selectivity for diverse microbial pathogens is still considerable. To distinguish between pathogens and viruses, Wang et al. employed a complex consisting of a cationic PT derivative functionalized with a quaternary ammonium group (**3**) and a barrel‐shaped macrocyclic molecule (i.e., cucurbit[7]uril (CB[7]))[^64^, ^65^] (Figure 5a).^[66]^ Target pathogens and viruses (i.e., tobacco mosaic virus [TMV], *Escherichia coli*, *Candida albicans*, and *Staphylococcus aureus*) possess negatively charged surfaces, whereby the PT derivative with a positively charged side chain (**3**) interacts with negatively charged analytes through electrostatic and hydrophobic reactions.^[61]^ The artificial receptor CB[7] possesses a hydrophobic cavity and a hydrophilic exterior, which plays a role in the suppression of inherent polymer aggregation by encapsulating the alkyl side chain moiety of **3**. Therefore, the complex of **3** and CB[7] (**3**·CB[7]) shows favorable electrostatic interactions with negatively charged analytes. The difference in both PT chemosensors with and without CB[7] provided a variation in fluorescence properties upon analyte detection, which further contributed to obtaining fluorescence response patterns depending on the size, shape, and surface characteristics of the pathogens and viruses (Figure 5b). Figure 5c illustrates that all the tested samples, as four clusters, were classified using LDA. They employed a self‐assembly approach to manipulate the side‐chain characteristics of PT derivatives using macrocyclic receptors. This approach enables detecting and discriminating viruses and microorganisms through multiple surface interactions between the charged fluorescent PT derivative and negatively charged analytes.

**FIGURE 6:**
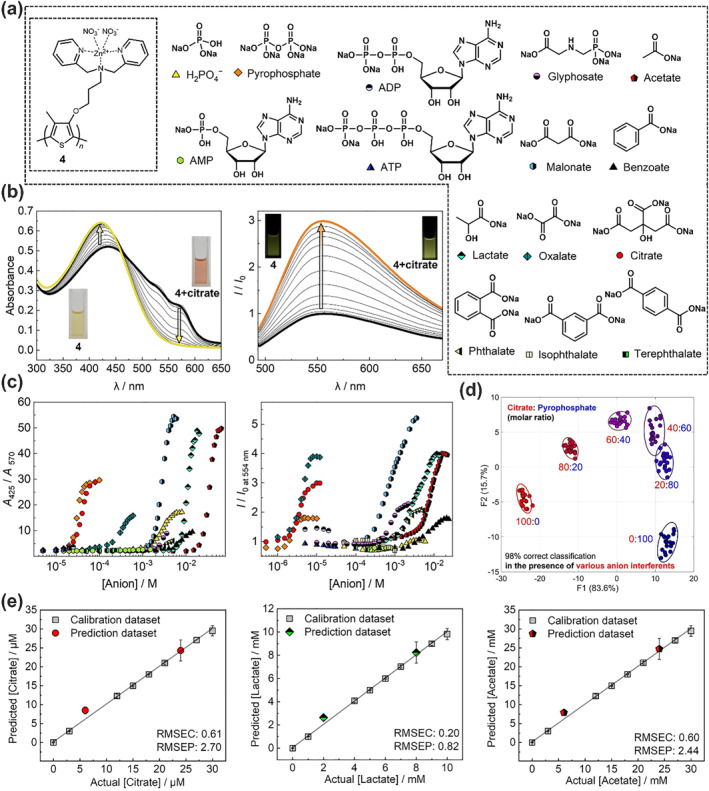
(a) Chemical structures of **4** and the analyte oxyanions. (b) (left) UV‐vis absorption spectra and (right) fluorescence spectra of **4** by adding citrate in aqueous methanol solution (methanol:water = 3:1, v/v) containing 2‐(*N*‐morpholino)ethanesulfonic acid and NaCl (pH = 5.5). [**4**] = 100 μM/unit, [citrate] = 0−10 μM for UV‐vis titration, [**4**] = 10 μM/unit, [citrate] = 0−12 μM for fluorescence titration. (c) (left) the relative absorbance and (right) fluorescence changes of **4** in the presence of the oxyanions at different concentration magnitudes. (d) Semi‐quantitative assessment with anion interferents (80 μM) for simultaneous discrimination of citrate and pyrophosphate in varied molar ratios. The mixtures of citrate and pyrophosphate was set as 80 μM. (e) SVM quantitative analysis for (left) citrate, (middle) lactate, and (right) acetate in mixtures. RMSEC, root mean square errors of calibration; RMSEP, root mean square errors of prediction. *Source*: Reproduced with permission from [29] 2023 Wiley‐VCH GmbH.

As discussed earlier, the optical alterations of functionalized PT derivatives are caused by the planarization of π‐conjugated systems and further polymer wire aggregation, which plays a pivotal role in obtaining a fingerprint‐like response pattern depending on analyte structures. However, these optical changes are accompanied by precipitation originating from the dominant polymer aggregations.[^31^, ^38^, ^49^, ^67^, ^68^, ^69^] Such unavoidable aggregation behavior causes fluctuations in the time‐dependent optical changes, leading to difficulties in quantitative analysis. Thus, Minami et al. proposed a PT‐based chemosensor for quantitative and qualitative detection based on random coil formation induced by analyte detection.^[29]^ Considering the polymer dynamics manipulated by the hydrophilicity of the analytes, an amphiphilic PT derivative functionalized with a zinc(II)‐dipicolylamine moiety (**4**) was employed for the pattern recognition of oxyanions (Figure 6a). Oxyanions are crucial markers in diagnostics and food analysis.[^70^, ^71^, ^72^, ^73^, ^74^, ^75^] Despite the advancements in chemical sensors for anions, the simultaneous discrimination of diverse anions remains challenging because of their structural variability.[^76^, ^77^, ^78^] In this sensing mechanism, anion exchange from hydrophobic nitrates (i.e., counter anions of the zinc(II) ion) to a hydrophilic oxyanion (i.e., citrate) induces blue shifts in the UV‐vis absorption spectra (Figure 6b, left) and fluorescence enhancement (Figure 6b, right). In addition, the PT chemosensor showed different magnitudes of optical response to 15 oxyanions at various concentration ranges in the selectivity test (Figure 6c). The response patterns were attributed to characteristics such as the binding affinity, hydrophilicity of the anions, and molecular geometry. In this regard, the colorimetric changes were caused by polymer dynamics in the presence of oxyanions, whereas fluorescence changes were induced by the molecular wire effect. Therefore, different response patterns were observed in the colorimetric and fluorescence profiles (Figure 6c). This unique sensing ability of the PT derivative was applied to a semi‐quantitative assay for the group categorization of pyrophosphate and citrate in mixtures of 14 anions (i.e., 13 oxyanions and chloride ions). Figure 6d illustrates the LDA results, showing 95% confidence ellipsoids of each cluster formed from the citrate and pyrophosphate mixtures. All clusters exhibited a molar ratio tendency, indicating the successful discrimination of various oxyanions at different concentrations within their mixtures. Furthermore, a quantitative assay targeting citrate, lactate, and acetate in the category of carboxylates was performed to evaluate the detection capabilities of **4** across various concentrations of oxyanions. In this assay, the unknown concentrations of the three oxyanions were predicted in their mixed solutions, and the discrimination power of **4** against structurally similar oxyanions at different concentrations was evaluated (Figure 6e). A single PT derivative with a dual response pattern allowed not only the simultaneous group categorization of phosphates and carboxylates but also the prediction of unknown concentrations of three diverse oxyanions within the micromolar to millimolar range.

Overall, the PT derivatives exhibited optical patterns derived from polymer dynamics and molecular wire effects in chemical sensing. These properties of PTs enable pattern recognition, albeit with a single chemosensor. Although PT‐based chemosensor arrays in solution media have many advantages in sensing applications, the requirement for large instruments to record optical responses causes difficulties in onsite chemical sensing. Keeping this in mind, the next section focuses on PT‐based chemosensor arrays using a solid support material for the development of onsite analytical devices.

## 4 APPROACHES TO ONSITE ANALYSIS USING POLYTHIOPHENE‐BASED CHEMOSENSOR ARRAY CHIPS

**FIGURE 7:**
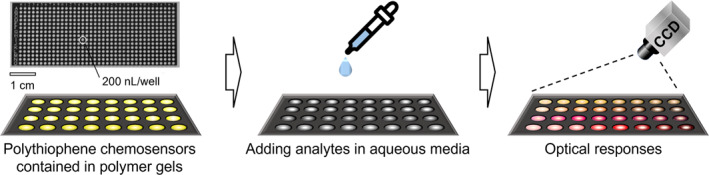
Schematic illustration of a solid‐state polythiophene‐based chemosensor array chip for onsite analysis.

Polymer gels of hydrophobic and hydrophilic moieties display both solid‐ and liquid‐like characteristics and have been widely used as solid support materials in various sensing applications.[^79^, ^80^] Since chemosensors and analytes are easily hydrated in aqueous media,^[81]^ the intrinsic nature of aquatic environments causes difficulty in effective molecular recognition. The hydrophobic and hydrophilic nature of polymer hydrogels plays a pivotal role in increasing the ability of chemosensors to interact robustly with analytes in water. Furthermore, these polymer gels contribute to suppressing fluorescence quenching caused by the self‐aggregation of fluorescent chemosensors in the solid state.[^82^, ^83^] These characteristics indicate the potential of polymer gels as solid‐state support materials for enhancing the practical effectiveness of chemosensor devices.[^80^, ^84^] In this approach, polymer gels embedded with PT chemosensors are applied to the sensing portions owing to the high absorbability of aqueous samples containing analytes.[^85^, ^86^] The PT chemosensor layers arrayed on the glass chip show optical responses upon the addition of analytes, which are rapidly captured using a recording apparatus (e.g., a charge‐coupled device (CCD) camera) (Figure 7). The obtained digital images are treated with imaging analysis techniques to extract the color intensities from the original images, which are further applied to pattern recognition (vide supra).

**FIGURE 8:**
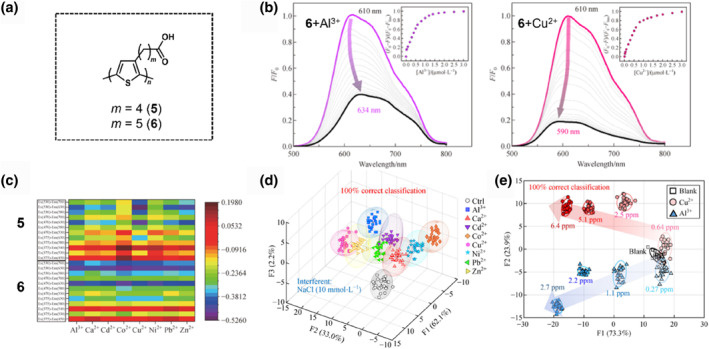
(a) Chemical structures of PT derivatives (**5** and **6**) for metal ion detection. (b) Fluorescence spectra (*λ*_ex_ = 480 nm) of **6** (10 μM/unit) upon increasing the concentrations of (left) Al^3+^ (right) Cu^2+^ ions. [Analyte] = 0−3 mM. (c) The heat map image of the array showing responses to various metal ions. (d) Qualitative analysis of eight different types of metal ions using LDA. (e) Semi‐quantitative analysis of Cu^2+^ and Al^3+^ ions using LDA. *Source*: Reproduced with permission from [69] 2021 Higher Education Press.

With the motive to design PT derivative‐based solid‐state chemosensor array chips, the Minami group selected two variants of carboxy‐group‐functionalized PT derivatives (**5** and **6**) for the detection of metal ions, such as copper(II) (Cu^2+^), zinc(II) (Zn^2+^), nickel(II) (Ni^2+^), cadmium(II) (Cd^2+^), aluminum(III) (Al^3+^), lead(II) (Pb^2+^), cobalt(II) (Co^2+^), and calcium(II) (Ca^2+^) ions (Figure 8a).^[69]^ In the chemosensor design, carboxy groups were introduced into the side chains of PT derivatives to detect metal ions. From the viewpoint of the linker chain length, a shorter alkyl chain (*m* = 3) promotes a doping effect on the PTs by metal ions, whereas a longer alkyl chain (*m* = 6) causes aggregation in polar solvents.[^87^, ^88^] Thus, appropriate linker lengths (i.e., *n*‐butyl carboxy [*m* = 4] and *n*‐pentyl carboxy‐functionalized PTs [*m* = 5]) were employed to evaluate the sensing ability of the metal ions. As shown in Figure 8b, the PT chemosensor exhibited different fluorescence profiles during the titration of metal ions (i.e., Al^3+^ (left) and Cu^2+^ ions (right)), even in the same polymer chemosensor. The difference in the fluorescence spectral change was attributed to the photophysical principle (e.g., spin‐orbital coupling) and polymer dynamics involving π‐conjugated systems and interchain interactions.[^89^, ^90^, ^91^, ^92^] In addition, the PT derivative **6** showed higher Stern‐Volmer quenching constants (*K*_SV_) for metal ions than the PT derivative **5**. The fluorescence response patterns of chemosensors **5** and **6** were obtained depending on the differences in the *K*_SV_s, although the PT chemosensors consisted of the same polymer backbone and recognition elements. This difference in the magnitudes of the optical responses can be ascribed to the stabilizing effect of the longer alkyl chains within the coordination complex.^[93]^ To facilitate practical onsite detection, an array device was implemented on a compact polyurethane hydrogel‐embedded glass chip. Commercially available polyurethanes (HydroMed D4) were selected for the construction of this device because of their ability to obtain appropriate sensing environments, which is attributed to both the hydrophobic and hydrophilic characteristics inherent in these polyurethanes.[^94^, ^95^] A 405‐well microarray chip comprising **5** and **6** was used for the qualitative and quantitative discrimination of metal ions in aqueous environments. The array chip was fabricated using an automated robotic dispenser with a volume of 0.2 μL per well. Metal ions in the buffer solutions were injected separately into the wells for chemical sensing. The fluorescence patterns of the microchip were recorded using a CCD camera equipped with UV, blue, and green light sources along with six color filters that provided red, blue, and green variations. Consequently, the heatmap in Figure 8c exhibits a fingerprint‐like response pattern upon metal ion recognition. The qualitative analysis data were subjected to LDA using the jackknife method, achieving accurate discrimination with a 100% correct classification rate for the eight types of metal ions (Figure 8d). Furthermore, the LDA canonical score plot in the semi‐quantitative detection of Al^3+^ and Cu^2+^ ions showed a concentration‐dependent cluster distribution, which suggested the contribution of the unique fluorescence profiles of the PT chemosensors (Figure 8e). This work presents an approach for fabricating chemosensor array chips by employing polymer gels, demonstrating high‐throughput sensing in combination with imaging analysis and pattern recognition techniques.

**FIGURE 9:**
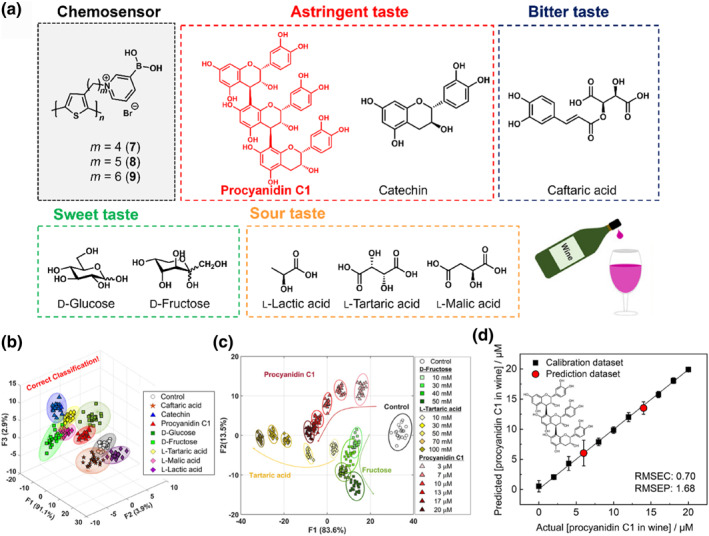
(a) Illustration of chemical structures encompassing PT‐based chemosensors (**7**, **8**, and **9**) and the target wine components. (b) Canonical sore plots derived from LDA offering qualitative analysis of eight wine components and control. (c) Semi‐quantitative assay of d‐fructose (square), l‐tartaric acid (diamond), and procyanidin C1 (triangle), using LDA. (d) The outcome of SVM regression analysis for procyanidin C1 in red wine. RMSEC, root mean square errors of calibration; RMSEP, root mean square errors of prediction. *Source*: Reproduced with permission from [31] 2020 Wiley‐VCH GmbH.

As described above, functionalized PT derivatives possess a wide detectable concentration range for various analytes with different molecular geometries. Wine components include sourness (l‐lactic acid, l‐tartaric acid, and l‐malic acid), bitterness (caftaric acid), sweetness (d‐glucose and d‐fructose), and astringent (procyanidin C1 and catechin), which are present at different concentrations.[^95^, ^96^, ^97^, ^98^] Therefore, a comprehensive evaluation of wine flavor requires the simultaneous and quantitative detection of various wine components (Figure 9a). To expand the potential of the array chip as an artificial tongue, fluorescent PT derivatives functionalized with 3‐pyridinium boronic acid (**7**, **8**, and **9**) were used for wine analysis.^[31]^ PT derivatives undergo a transformative zwitterionic process upon binding to wine components containing diols, leading to self‐aggregation and subsequent fluorescence quenching. For example, the addition of fructose caused moderate fluorescence quenching at millimolar (mM) concentrations, whereas procyanidin C1 demonstrated drastic fluorescence quenching at submicromolar (sub‐µM) levels owing to its multiple binding sites. In contrast, l‐tartaric acid induced a sigmoidal response with a redshift at millimolar levels, attributed to alterations in the polarity surrounding the PT backbone. These distinctive fluorescence profiles were observed upon the addition of analytes containing carboxy groups, such as sour and bitter components, which can be explained by multiple interactions between boronic acid of the PT derivatives and the *α*‐hydroxy carboxylate groups of the wine components.^[100]^ This intricate interplay between the PT derivatives and wine constituents underscores the potential for discriminating multiple taste components at different concentrations. Therefore, the PT derivatives contained in the polymer gel material were applied as a chemosensor array chip for the simultaneous detection of wine components using imaging analysis and pattern recognition techniques. The dataset employed for pattern recognition encompassed fluorescence intensity information across various excitation wavelengths, culminating in a comprehensive 3840 datasets after careful data pre‐processing. According to actual concentrations in the wine components, each concentration was set at 10 μM for assessing bitterness and astringency, while sourness and sweetness evaluations were conducted at 30 mM. This methodology combined with LDA resulted in classifying nine clusters, comprising eight analytes and a control group (Figure 9b). Moreover, the sensing system performed semi‐quantitative detection of fructose as sweetness, l‐tartaric acid as sourness, and procyanidin C1 as astringency at different concentrations (Figure 9c). The distribution of each cluster was visualized using LDA, and the concentration dependency suggested favorable recognition abilities of the chemosensor array chip against the analyte structures and their concentrations. SVM analysis was employed to predict the unknown concentrations of procyanidin C1 in red wine, specifically in Cabernet Sauvignon. This application demonstrated the practicality of fluorescent artificial tongue based on PT derivatives for authentic wine analysis (Figure 9d). In essence, the integration of PT‐based fluorescent chemosensors with imaging analysis and pattern‐recognition techniques has culminated in the establishment of authentic artificial tongues.

**FIGURE 10:**
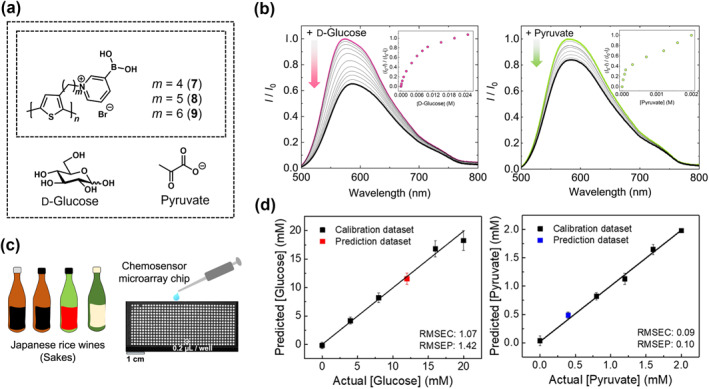
(a) Depiction of the chemical structures of PT‐based chemosensors (**7**, **8**, and **9**) and the analytes in sake analysis. (b) Fluorescence spectra (λ_ex_ = 460 nm) of (left) **8** with the addition of glucose and (right) **7** with the addition of pyruvate in a mixture of phosphate buffer (pH 7.0) and methanol (1:9, v/v) solution. [**7**] = [**8**] = 10 μM/unit, [glucose] = 0−25 mM, and [pyruvate] = 0−2 mM. (c) An image showcasing the microarray chip for chemosensor applications. (d) SVM outcomes depicting the concentrations of (left) glucose and (right) pyruvate in a mixture of a Japanese rice wine sample and phosphate buffer (pH = 7.0). RMSEC, root mean square errors of calibration; RMSEP, root mean square errors of prediction. *Source*: Reproduced with permission from [101] 2021 The Society of Polymer Science.

The application of the same PT‐based chemosensors (**7**, **8**, and **9**) was extended to the analysis of Japanese rice wine, commonly known as sake, to discern the presence of glucose and pyruvate (Figure 10a).^[101]^ The identification of glucose is pivotal for evaluating the sweetness profiles of different sakes, whereas the detection of pyruvate serves as a valuable indicator for estimating the fermentation of these beverages. Similar to the aforementioned example, functionalized PT‐based chemosensors undergo a transformative shift into zwitterionic forms upon interaction with analytes in sake samples. This process led to polymer aggregation and subsequently induced fluorescence quenching (Figure 10b). A chemosensor microarray composed of the PT derivatives and polymer gel was fabricated on a glass chip substrate, and the fluorescence responses were captured using a CCD camera (Figure 10c). The PT‐based chemosensor array chips were utilized for quantitative analysis to assess their suitability as onsite sake detection tools. The unknown concentrations of glucose and pyruvate within the sake sample were accurately quantified using the SVM (Figure 10d). This study demonstrated the potential of accurate and user‐friendly onsite food analysis using a PT‐based fluorescence chemosensor array chip. Hence, onsite chemosensor array devices utilizing PT derivatives will be highly effective for rapidly detecting diverse analytes by combining imaging analysis and pattern recognition techniques.

## 5 CONCLUSION AND FUTURE PERSPECTIVES

Chemosensor array systems allow simultaneous qualitative and quantitative detection of various analytes in combination with powerful pattern recognition methods. Conventional arrays require various chemosensors with different optical properties to obtain fingerprint‐like response profiles for pattern recognition. However, this approach cannot avoid contaminating noise datasets derived from low‐contribution chemosensors, which decreases the accuracy of the sensing results. This review focuses on PT as a chemosensor backbone for pattern recognition owing to its unique polymer behavior. The inherent polymer properties include molecular wire effects and dynamic structural changes in π‐conjugated systems, which induce optical changes upon the analyte detection at a functionalized side chain. The magnitude of the optical response depends on the analyte structure and concentration. Thus, various optical response profiles contribute to obtaining an information‐rich dataset, even for a single chemosensor. Functionalized PT‐derived chemosensors have emerged as highly promising platforms for pattern recognition through various demonstrations in environmental monitoring, medical diagnostics, and food analysis. In addition, using polymer gels facilitates the realization of portable sensor chips, allowing high‐throughput sensing combined with imaging analysis techniques. However, unlike solution‐state chemical sensors, several chemosensors are required for solid‐state pattern recognition using polymer gels. The requirement for chemosensor preparation in solid‐state array devices is attributed to the suppression of polymer dynamics in the solid support environment, which are concerns for the development of practical chemosensor array devices. In this regard, the inherent aggregation behavior of π‐conjugated polymers is a bottleneck in developing solid‐state chemosensor devices. Therefore, this review focused on polymer gels as solid‐state support materials to disperse π‐conjugated polymer wires. Paper substrates are promising candidates for ordinary solid‐state materials. Paper‐based analytical devices have been extensively developed owing to their high processability.[^95^, ^102^] In addition, the optical responses of paper substrates in chemical sensing can be recorded using portable cameras.^[103]^ However, applications for paper‐based π‐conjugated polymer sensor devices are still rare. This is attributed to the research trend considering the inherent aggregation behaviors of π‐conjugated polymers in the solid states. Meanwhile, the significant contribution of paper fiber structures to the dispersion of π‐conjugated polymers was revealed by the demonstration of highly sensitive detection at ppb levels on a paper‐based chemosensor array device very recently.^[104]^ We believe the approaches summarized in this review to provide intramolecular wire effects in solid states could facilitate the development of π‐conjugated polymer‐based chemosensors in solid states for onsite chemical detection.

## CONFLICT OF INTEREST STATEMENT

The authors declare no conflicts of interest.

## References

[1] K. J. Albert , N. S. Lewis , C. L. Schauer , G. A. Sotzing , S. E. Stitzel , T. P. Vaid , D. R. Walt , Chem. Rev. 2000, 100, 2595. 11749297 10.1021/cr980102w

[2] P. Anzenbacher, Jr. , P. Lubal , P. Buček , M. A. Palacios , M. E. Kozelkova , Chem. Soc. Rev. 2010, 39, 3954. 20820464 10.1039/b926220m

[3] Z. Li , J. R. Askim , K. S. Suslick , Chem. Rev. 2019, 119, 231. 30207700 10.1021/acs.chemrev.8b00226

[4] K. L. Diehl , E. V. Anslyn , Chem. Soc. Rev. 2013, 42, 8596. 23999658 10.1039/c3cs60136f

[5] Y. Sasaki , T. Minami , ChemNanoMat 2023, 10, e202300335.

[6] Y. Sasaki , R. Kubota , T. Minami , Coord. Chem. Rev. 2021, 429, 213607.

[7] L. Mitchell , E. J. New , C. S. Mahon , ACS Appl. Polym. Mater. 2021, 3, 506.

[8] C. Li , G. Shi , ACS Appl. Mater. Interfaces 2013, 5, 4503. 23429878 10.1021/am400009d

[9] Y. Sasaki , X. Lyu , W. Tang , H. Wu , T. Minami , Bull. Chem. Soc. Jpn. 2021, 94, 2613.

[10] M. Ikeda , R. Ochi , I. Hamachi , Lab Chip 2010, 10, 3325. 20862441 10.1039/c004908e

[11] S. Rana , A. K. Singla , A. Bajaj , S. G. Elci , O. R. Miranda , R. Mout , B. Yan , F. R. Jirik , V. M. Rotello , ACS Nano 2012, 6, 8233. 22920837 10.1021/nn302917e PMC3603354

[12] S. Rana , Y.‐C. Yeh , V. M. Rotello , Curr. Opin. Chem. Biol. 2010, 14, 828. 21035376 10.1016/j.cbpa.2010.10.001 PMC2997876

[13] Y. Liu , T. Minami , R. Nishiyabu , Z. Wang , P. Anzenbacher, Jr. , J. Am. Chem. Soc. 2013, 135, 7705. 23656505 10.1021/ja4015748

[14] Y. Sasaki , S. Kojima , V. Hamedpour , R. Kubota , S. Takizawa , I. Yoshikawa , H. Houjou , Y. Kubo , T. Minami , Chem. Sci. 2020, 11, 3790.

[15] Y. Sasaki , K. Ohshiro , Q. Zhou , X. Lyu , W. Tang , K. Okabe , S. Takizawa , T. Minami , Chem. Commun. 2023, 59, 7747. 10.1039/d3cc00949a 37272870

[16] A. C. Sedgwick , J. T. Brewster , T. Wu , X. Feng , S. D. Bull , X. Qian , J. L. Sessler , T. D. James , E. V. Anslyn , X. Sun , Chem. Soc. Rev. 2021, 50, 9. 33169731 10.1039/c9cs00538b

[17] Y. Wang , T. Michinobu , Bull. Chem. Soc. Jpn. 2017, 90, 1388.

[18] T. M. Swager , Acc. Chem. Res. 1998, 31, 201.

[19] H. N. Kim , Z. Guo , W. Zhu , J. Yoon , H. Tian , Chem. Soc. Rev. 2011, 40, 79. 21107482 10.1039/c0cs00058b

[20] G. Zhang , X. Fu , D. Zhou , R. Hu , A. Qin , B. Z. Tang , Smart Mol. 2023, 1, e20220008. 10.1002/smo.20220008 PMC12118199 40625652

[21] S. Sabury , G. S. Collier , M. N. Ericson , S. M. Kilbey II , Polym. Chem. 2020, 11, 820.

[22] M.‐P. Plante , E. Bérubé , L. Bissonnette , M. G. Bergeron , M. Leclerc , ACS Appl. Mater. Interfaces 2013, 5, 4544. 23521757 10.1021/am400162h

[23] D. Cheng , Y. Li , J. Wang , Y. Sun , L. Jin , C. Li , Y. Lu , Chem. Commun. 2015, 51, 8544. 10.1039/c5cc01713k 25894335

[24] C. Li , M. Numata , M. Takeuchi , S. Shinkai , Angew. Chem. Int. Ed. 2005, 117, 6529. 10.1002/anie.200501823 16173002

[25] G. Sinsinbar , A. Palaniappan , U. H. Yildiz , B. Liedberg , ACS Sens. 2022, 7, 686. 35226461 10.1021/acssensors.1c02476

[26] T. Minami , R. Nishiyabu , M. Iyoda , Y. Kubo , Chem. Commun. 2010, 46, 8603. 10.1039/c0cc03179h 20890502

[27] C.‐Y. Kuo , Y.‐C. Huang , C.‐Y. Hsiow , Y.‐W. Yang , C.‐I. Huang , S.‐P. Rwei , H.‐L. Wang , L. Wang , Macromolecules 2013, 46, 5985.

[28] T. Minami , Y. Kubo , Chem. Asian J. 2010, 5, 605. 20095000 10.1002/asia.200900444

[29] Y. Sasaki , K. Ohshiro , K. Okabe , X. Lyu , K. Tsuchiya , A. Matsumoto , S. Takizawa , T. Minami , Chem. Asian J. 2023, 18, e202300372. 37309739 10.1002/asia.202300372

[30] Y. L. Pak , Y. Wang , Q. Xu , Coord. Chem. Rev. 2021, 433, 213745.

[31] Y. Sasaki , S. Ito , Z. Zhang , X. Lyu , S. Takizawa , R. Kubota , T. Minami , Chem. Eur. J. 2020, 26, 16236. 32633434 10.1002/chem.202002262

[32] T. Minami , N. A. Esipenko , B. Zhang , M. E. Kozelkova , L. Isaacs , R. Nishiyabu , Y. Kubo , P. Anzenbacher, Jr. , J. Am. Chem. Soc. 2012, 134, 20021. 23194337 10.1021/ja3102192

[33] L. H. Hamel , Knowledge Discovery with Support Vector Machines, John Wiley & Sons, Inc 2011.

[34] L. Zhu , S. H. Shabbir , E. V. Anslyn , Chem. Eur. J. 2007, 13, 99. 17066491 10.1002/chem.200600402

[35] S. C. McCleskey , P. N. Floriano , S. L. Wiskur , E. V. Anslyn , J. T. McDevitt , Tetrahedron 2003, 59, 10089.

[36] A. M. Zain , H. Haron , S. N. Qasem , S. Sharif , Appl. Math. Model. 2012, 36, 1477.

[37] M. Jalali‐Heravi , M. Arrastia , F. A. Gomez , Anal. Chem. 2015, 87, 3544. 25651407 10.1021/ac504863y

[38] Z. Yao , X. Feng , W. Hong , C. Li , G. Shi , Chem. Commun. 2009, 4696. 10.1039/b904975d 19641813

[39] J. M. Berg , J. L. Tymoczko , L. Stryer , Biochemistry 2002, 433.

[40] S. E. Schneider , S. N. O’Nei , E. V. Anslyn , J. Am. Chem. Soc. 2000, 122, 542.

[41] A. Buryak , A. Pozdnoukhov , K. Severin , Chem. Commun. 2007, 2366. 10.1039/b705250b 17844748

[42] J. Y. Kwon , N. J. Singh , H. N. Kim , S. K. Kim , K. S. Kim , J. Yoon , J. Am. Chem. Soc. 2004, 126, 8892. 15264809 10.1021/ja0492440

[43] A. Ojida , I. Takashima , T. Kohira , H. Nonaka , I. Hamachi , J. Am. Chem. Soc. 2008, 130, 12095. 18700758 10.1021/ja803262w

[44] A. Ojida , H. Nonaka , Y. Miyahara , S. I. Tamaru , K. Sada , I. Hamachi , Angew. Chem. Int. Ed. 2006, 45, 5518. 10.1002/anie.200601315 16847978

[45] P. P. Neelakandan , M. Hariharan , D. Ramaiah , J. Am. Chem. Soc. 2006, 128, 11334. 16939239 10.1021/ja062651m

[46] S. Wang , Y.‐T. Chang , J. Am. Chem. Soc. 2006, 128, 10380. 16895399 10.1021/ja063733d

[47] C. Li , M. Numata , M. Takeuchi , S. Shinkai , Chem. Asian J. 2006, 1, 95. 17441043 10.1002/asia.200600039

[48] M. Derakhshesh , M. R. Gray , G. P. Dechaine , Energy Fuel. 2013, 27, 680.

[49] M. S. Maynor , T. L. Nelson , C. O’Sulliva , J. J. Lavigne , Org. Lett. 2007, 9, 3217. 17637024 10.1021/ol071065a

[50] H. M. Wallace , R. Caslake , Eur. J. Gastroenterol. Hepatol. 2001, 13, 1033. 11564951 10.1097/00042737-200109000-00006

[51] C. Ruiz‐Capillas , A. M. Herrero , Foods 2019, 8, 62. 30744001 10.3390/foods8020062 PMC6406683

[52] Y. Özogul , F. Özogul , Biogenic Amines Formation, Toxicity, Regulations in Food. Biogenic Amines in Food: Analysis, Occurrence and Toxicity, The Royal Society of Chemistry 2019, 1–17.

[53] H. Wolrath , U. Forsum , P.‐G. Larsson , H. Borén , J. Clin. Microbiol. 2001, 39, 4026. 11682525 10.1128/JCM.39.11.4026-4031.2001 PMC88482

[54] L. Luo , J.‐Y. Yang , Z.‐L. Xiao , D.‐P. Zeng , Y.‐J. Li , Y.‐D. Shen , Y.‐M. Sun , H.‐T. Lei , H. Wang , Z.‐L. Xu , RSC Adv. 2015, 5, 78833.

[55] N. G. Sanceda , E. Suzuki , M. Ohashi , T. Kurata , J. Agric. Food Chem. 1999, 47, 3596. 10552691 10.1021/jf9812174

[56] J. Rosier , C. Van Peteghem , Z. Lebensm. Unters. Forsch. 1988, 186, 25. 3354263 10.1007/BF01027175

[57] F. Feng , F. He , L. An , S. Wang , Y. Li , D. Zhu , Adv. Mater. 2008, 20, 2959.

[58] Y. Liu , K. Ogawa , K. S. Schanze , J. Photochem. Photobiol. C 2009, 10, 173.

[59] S. W. Thomas , G. D. Joly , T. M. Swager , Chem. Rev. 2007, 107, 1339. 17385926 10.1021/cr0501339

[60] X. Duan , L. Liu , F. Feng , S. Wang , Acc. Chem. Res. 2010, 43, 260. 19954139 10.1021/ar9001813

[61] H. Yuan , Z. Liu , L. Liu , F. Lv , Y. Wang , S. Wang , Adv. Mater. 2014, 26, 4333. 24737340 10.1002/adma.201400636

[62] A. Duarte , A. Chworos , S. F. Flagan , G. Hanrahan , G. C. Bazan , J. Am. Chem. Soc. 2010, 132, 12562. 20731391 10.1021/ja105747b

[63] C. Zhu , Q. Yang , L. Liu , S. Wang , J. Mater. Chem. 2011, 21, 7905.

[64] J. Lagona , P. Mukhopadhyay , S. Chakrabarti , L. Isaacs , Angew. Chem. Int. Ed. 2005, 44, 4844. 10.1002/anie.200460675 16052668

[65] E. Pazos , P. Novo , C. Peinador , A. E. Kaifer , M. D. García , Angew. Chem. Int. Ed. 2019, 58, 403. 10.1002/anie.201806575 29978946

[66] H. Bai , H. Lu , X. Fu , E. Zhang , F. Lv , L. Liu , S. Wang , Biomacromolecules 2018, 19, 2117. 29634899 10.1021/acs.biomac.8b00336

[67] T. L. Nelson , C. O’Sullivan , N. T. Greene , M. S. Maynor , J. J. Lavigne , J. Am. Chem. Soc. 2006, 128, 5640. 16637623 10.1021/ja060589n

[68] T. L. Nelson , I. Tran , T. G. Ingallinera , M. S. Maynor , J. J. Lavigne , Analyst 2007, 132, 1024. 17893806 10.1039/b708583d

[69] Y. Sasaki , X. Lyu , Z. Zhang , T. Minami , Front. Chem. Sci. Eng. 2022, 16, 72.

[70] Q. Zhang , Y. Zhou , M. Ahmed , N. M. Khashab , W. Han , H. Wang , Z. A. Page , J. L. Sessler , J. Mater. Chem. A 2022, 10, 15297.

[71] M. S. Taylor , Coord. Chem. Rev. 2020, 413, 213270.

[72] E. J. O’Neil , B. D. Smith , Coord. Chem. Rev. 2006, 250, 3068.

[73] X. Wu , A. M. Gilchrist , P. A. Gale , Chem 2020, 6, 1296.

[74] D. Yang , J. Zhao , X.‐J. Yang , B. Wu , Org. Chem. Front. 2018, 5, 662.

[75] P. A. Gale , E. N. W. Howe , X. Wu , Chem 2016, 1, 351.

[76] A. Borissov , I. Marques , J. Y. C. Lim , V. Félix , M. D. Smith , P. D. Beer , J. Am. Chem. Soc. 2019, 141, 4119. 30730716 10.1021/jacs.9b00148

[77] J. H. Oh , B. P. Hay , V. M. Lynch , H. Li , J. L. Sessler , S. K. Kim , J. Am. Chem. Soc. 2022, 144, 16996. 36074582 10.1021/jacs.2c06284

[78] W. Liu , A. G. Oliver , B. D. Smith , J. Org. Chem. 2019, 84, 4050. 30827107 10.1021/acs.joc.9b00042

[79] P. Anzenbacher, Jr. , Y. Liu , M. E. Kozelkova , Curr. Opin. Chem. Biol. 2010, 14, 693. 20817542 10.1016/j.cbpa.2010.08.011

[80] O. S. Wolfbeis , J. Mater. Chem. 2005, 15, 2657.

[81] M. J. Langton , C. J. Serpell , P. D. Beer , Angew. Chem. Int. Ed. 2016, 55, 1974. 10.1002/anie.201506589 PMC4755225 26612067

[82] S. Zahn , T. M. Swager , Angew. Chem. Int. Ed. 2002, 41, 4225. 10.1002/1521-3773(20021115)41:22<4225::AID-ANIE4225>3.0.CO;2-3 12434346

[83] Y. Sasaki , K. Asano , T. Minamiki , Z. Zhang , S. Takizawa , R. Kubota , T. Minami , Chem. Eur. J. 2020, 26, 14525. 32803889 10.1002/chem.202003529

[84] Y. Liu , M. A. Palacios , P. Anzenbacher , Chem. Commun. 2010, 46, 1860. 10.1039/b925506k 20198233

[85] J. A. Bouwstra , M. A. Salomons‐de Vries , J. C. Van Miltenburg , Thermochim. Acta 1995, 248, 319.

[86] G. G. Ferrer , M. M. Pradas , J. L. G. Ribelles , M. S. Sánchez , Polymer 2004, 45, 6207.

[87] M. B. Inoue , E. F. Velazquez , M. Inoue , Synth. Met. 1988, 24, 223.

[88] T. Minami , Y. Kubo , Supramol. Chem. 2011, 23, 13.

[89] Y. Chen , K.‐Y. Pu , Q.‐Li. Fan , X.‐Y. Qi , Y.‐Q. Huang , X.‐M. Lu , W. Huang , J. Polym. Sci. Polym. Chem. 2009, 47, 5057.

[90] J. You , J. Kim , T. Park , B. Kim , E. Kim , Adv. Funct. Mater. 2012, 22, 1417.

[91] X. Wang , J. Zhao , C. Guo , M. Pei , G. Zhang , Sens. Actuators B Chem. 2014, 193, 157.

[92] S. C. Rasmussen , S. J. Evenson , C. B. McCausland , Chem. Commun. 2015, 51, 4528. 10.1039/c4cc09206f 25622008

[93] T. Bala , B. L. V. Prasad , M. Sastry , M. U. Kahaly , U. V. Waghmare , J. Phys. Chem. A 2007, 111, 6183. 17585841 10.1021/jp067906x

[94] B. J. Müller , T. Rappitsch , C. Staudinger , C. Rüschitz , S. M. Borisov , I. Klimant , Anal. Chem. 2017, 89, 7195. 28585806 10.1021/acs.analchem.7b01373

[95] Y. Sasaki , X. Lyu , W. Tang , H. Wu , T. Minami , J. Photochem. Photobiol. C 2022, 51, 100475.

[96] R. A. Arnold , A. C. Noble , V. L. Singleton , J. Agric. Food Chem. 1980, 28, 675.

[97] N. Martin , Food Qual. Prefer. 2002, 13, 295.

[98] J. M. Ricardo‐da‐Silva , J.‐P. Rosec , M. Bourzeix , J. Mourgues , M. Moutounet , Vitis ‐ J. Grapevine Res. 2015, 31, 55.

[99] J. Han , M. Bender , K. Seehafer , U. H. F. Bunz , Angew. Chem. Int. Ed. 2016, 55, 7689. 10.1002/anie.201602385 27257821

[100] C. W. Gray , T. A. Houston , J. Org. Chem. 2002, 67, 5426. 12126445 10.1021/jo025876y

[101] X. Lyu , A. Matsumoto , T. Minami , Polym. J. 2021, 53, 1287.

[102] F. Ghasemi , N. Fahimi‐Kashani , A. Bigdeli , A. H. Alshatteri , S. Abbasi‐Moayed , S. H. Al‐Jaf , M. Y. Merry , K. M. Omer , M. R. Hormozi‐Nezhad , Anal. Chim. Acta 2023, 1238, 340640. 36464453 10.1016/j.aca.2022.340640

[103] J. R. Askim , K. S. Suslick , Anal. Chem. 2015, 87, 7810. 26177346 10.1021/acs.analchem.5b01499

[104] Y. Sasaki , X. Lyu , T. Kawashima , Y. Zhang , K. Ohshiro , K. Okabe , K. Tsuchiya , T. Minami , RSC Adv. 2024, 14, 5159. 38332791 10.1039/d3ra08429a PMC10851342

